# Red Raspberry Extract Protects the Skin against UVB-Induced Damage with Antioxidative and Anti-inflammatory Properties

**DOI:** 10.1155/2019/9529676

**Published:** 2019-01-06

**Authors:** Pei-Wen Wang, Yu-Chen Cheng, Yu-Chiang Hung, Chih-Hung Lee, Jia-You Fang, Wen-Tai Li, Yun-Ru Wu, Tai-Long Pan

**Affiliations:** ^1^Department of Medical Research, China Medical University Hospital, China Medical University, Taichung, Taiwan; ^2^Department of Chinese Medicine, College of Medicine, Kaohsiung Chang Gung Memorial Hospital and Chang Gung University, Kaohsiung, Taiwan; ^3^Department of Dermatology, College of Medicine, Kaohsiung Chang Gung Memorial Hospital and Chang Gung University, Kaohsiung, Taiwan; ^4^Pharmaceutics Laboratory, Graduate Institute of Natural Products, Chang Gung University, Taoyuan, Taiwan; ^5^National Research Institute of Chinese Medicine, Ministry of Health and Welfare, Taipei, Taiwan; ^6^School of Traditional Chinese Medicine, Chang Gung University, Taoyuan, Taiwan; ^7^Liver Research Center, Chang Gung Memorial Hospital, Taoyuan, Taiwan; ^8^Chinese Herbal Medicine Research Team, Healthy Aging Research Center, Chang Gung University, Taoyuan, Taiwan; ^9^Research Center for Chinese Herbal Medicine and Research Center for Food and Cosmetic Safety, College of Human Ecology, Chang Gung University of Science and Technology, Taoyuan, Taiwan

## Abstract

Extensive exposure to UVB (280–320 nm) is the major risk responsible for various skin injuries. Numerous reports have shown that natural products could demonstrate photochemopreventive efficacy against UVB damage. We investigated the preventive effects and associated molecular mechanisms of red raspberry extract upon UVB-caused damage in human epidermal keratinocytes and a nude mouse model. The protein profiles and immunohistological study on a nude mouse skin indicated that red raspberry extract could prevent UVB-caused cell death and protect the skin against UVB-exposed injury manifested by wrinkling, scaling, tanning, and water loss as well as epidermal thickening. In addition, red raspberry extract application effectively abolished oxidative damage in DNA and attenuated the carbonylation level of proteins, which attributed to the activation of SOD, Nrf2 and its target genes, and HO-1. Red raspberry extract also altered the cells' apoptotic signaling pathways including caspase-3 as well as the inflammatory cascade such as c-jun and attenuated UVB-induced activation of NF-*κ*B and COX-2. Red raspberry extract could alleviate direct photodamage to the skin caused by UVB exposure through the ROS scavenger and protection against inflammatory responses, which may allow the development of novel strategies in protecting the skin subjected to UVB radiation.

## 1. Introduction

Photon energy, especially ultraviolet B (UVB) radiation, induces many deleterious effects including deoxyribonucleic acid (DNA) and protein damage, oxidative stress, inflammation, and carcinogenesis. Previous studies have suggested that these events are mainly caused by reactive oxygen species (ROS), which would eventually result in various skin diseases [1–3]. Application of antioxidants should therefore be the effective strategy for photoprotection of the skin [4–6].

Compelling evidence showed that berry fruits possess antioxidative, anti-inflammatory, and anticarcinogenic properties because berries contain large amounts of phytochemicals, including flavonoids, tannins, stillbenoids, phenolic acids, lignans, triterpenes, and sterols [7]. The dietary consumption of whole fruits could reduce ROS, which have been implicated in UVB-caused problems [8–10]. The traditional Chinese medicine book “Essential of Materia Medica” has descripted that raspberry could exhibit the effect of moistening the skin and reducing the redness as well as swelling of the skin. As expected, several bioactive constituents, including polyphenolic compounds, antioxidants, vitamins, and minerals, have been extracted from red raspberries (*Rubus idaeus*) [11–13], while the effects and molecular mechanisms of red raspberry on skin photodamage have not been reported. Herein, UVB-exposed hairless mice and keratinocyte models were applied to investigate the protective effect of ethanol extract of red raspberry (RBE) on a photodamaged skin.

ROS generation due to UVB radiation would disturb the normal redox balance and lead to highly oxidative stress, which subsequently promotes the carbonylation of specific groups of proteins and results in physiological dysfunction [14–16]. When carbonyl groups form, they can react with 2,4-dinitrophenylhydrazine (DNP) and are detected by two-dimensional electrophoresis (2-DE) oxyblotting; therefore, we utilized redox proteomics to prove our hypothesis concerning the anti-UVB effect of red raspberry.

UVB could also elicit acute inflammatory skin problems such as erythema and cell apoptosis. UVB-caused promotion of proinflammatory enzymes and the subsequent activation of an associated signaling pathway such as cyclooxygenase-2 (COX-2) would in turn trigger the production of specific inflammatory mediators including prostaglandins (PGs) and various cytokines. COX-2 cascades mediate the inflammatory process and cause pain, edema, cell growth, and tumor progression [17, 18]. It has been implied that inflammation plays a pivotal role in the pathogenesis of skin diseases under UVB exposure [19].

We performed an immunohistological investigation and established the redox proteome profiles on a nude mouse skin to verify the hypothesis that RBE could attenuate the oxidative stress caused by UVB and protect the skin from photoinjury. In addition, the associated molecular mechanisms would provide the clinical and commercial utility of herbal intervention in UVB prevention on the skin.

## 2. Materials and Methods

### 2.1. Preparation of Red Raspberry Ethanolic Extract

Commercial dry powder of raspberry was purchased and authenticated by a traditional Chinese medicine dispensary (local pharmaceutical company, Taiwan). The ethanol-extracted solution was then concentrated to give brown syrup. The filtered and sterile extract was stored at −80°C for use in all subsequent experiments. The concentration used in each experiment was calculated based upon the dry weight of the extract which was resuspended in normal saline.

### 2.2. Cell Viability

HaCaT cells (5 × 10^4^) were seeded in 24-well plates for 24 hours (h). UVB radiation (0 or 100 mJ/cm^2^) was exposed to the cells after treating with various concentrations (0, 62.5, 125, 250, 500, and 1000 *μ*g/mL) of RBE and incubated for 48 h. Isopropanol solution mixed with tetrazolium salt was then added to the wells and incubated for additional 4 h at 37°C [20]. The optical density of the dissolved material was measured spectrophotometrically at 570 nm, and assays were performed in triplicate.

### 2.3. Western Blot Analysis

HaCaT cells were pretreated with 200 *μ*g/mL RBE and exposed to 100 mJ/cm^2^ UVB radiation. The protein derived from the treatment was isolated using 1x cell lysis buffer (Cell Signaling), and the concentration was measured using the Bradford Protein Assay Kit (AMRESCO). Protein lysates were evaluated with Western blot analyses as previously described [20, 21]. Western blot analysis was performed using the specific antibodies: PARP, caspase-3 (DAKO), catalase (Bioss), Cu/ZnSOD (ABBIOTEC), GAPDH, MnSOD, Nrf2, HO-1, *β*-actin, phos-p38, p38, c-Jun, NF*κ*Bp65, and NF*κ*Bp50 (Santa Cruz). The levels of GAPDH or *β*-actin were used as the internal loading control. Densitometric analyses of scanned images were performed using GeneTools software (Syngene, UK).

### 2.4. siRNA and p-38 Kinase Inhibitor Administration

HaCaT cells were plated onto 24-well plates (2 × 10^4^ cells/well), maintained in antibiotic-free medium for 6 h, and transfected with a mixture containing Opti-MEM, 8 *μ*L/well Lipofectamine 2000 (Invitrogen, San Diego, CA) and 0.5 *μ*g/well a mixture of three Nrf2 siRNAs. At 24 h post transfection, cells were exposed to UVB and treated with 200 *μ*g/mL RBE dissolved in medium for another 24 h. Then, the cells were harvested for Western blot analyses [22]. HaCaT cells were preincubated with or without SB203580 for 1 h, irradiated with UVB, and then treated with or without RBE for 6 h. The cells were harvested for Western blot analyses [20].

### 2.5. Assessment for Generation of Intracellular ROS

HaCaT cells were seeded in a slide chamber, grown to 60% confluence, and cultured in DMEM medium overnight. Cells were then incubated with or without RBE for 6 h and irradiated by 100 mJ/cm^2^ UVB [23]. Carboxy-H2DCFDA (2 *μ*M, dissolved in PBS) was added to the wells and incubated for 30 min at 37°C. To terminate the reaction, the cells were washed with PBS twice. Next, 500 *μ*L culture medium was added to each well and incubated for 20 min at 37°C. The cells were observed and photographed using a fluorescent microscope (Olympus BX51) with a DP72 PhotoImage system [21].

### 2.6. Constitutions Analysis with HPLC

A high-performance liquid chromatographic (Shimadzu SCL-10A VP) coupled with an SPD-M10A VP diode array detector was performed for the qualitative determination of compounds in the RBE [24].

### 2.7. Animals

Female nude mice (ICR-Foxn/nu strain) were purchased from Taiwan's National Laboratory Animal Center (Taipei). The laboratory diet and water were given ad libitum before experiments. The mice were treated according to the Ethical Guidelines of the Animal Center, and the experimental protocol was reviewed and approved by the Institutional Animal Care and Use Committee of Kaohsiung Chang Gung Memorial Hospital (2017081401). The mice were randomly divided into three groups (CTL, −/UVB, and RBE/UVB) of five mice each. 750 *μ*g/mL RBE was applied on the dorsal region of the nude mice in the RBE/UVB group. The daily standard erythema dose of UVB for the human skin is more than 25 mJ/cm^2^. A Bio-Sun system illuminator (Vilber Lourmat, Marne-la-Vallée, France) was applied to generate UVB radiation which was utilized to irradiate the peak wavelengths of 312 nm. The distance between the nude mice and the lamps was 10 cm, and the spectral irradiance was 30 mJ/cm^2^ for UVB in the dorsal region of the mouse once a day for five days. Hence, the total UVB dose received by each mouse during the irradiation course was 150 mJ/cm^2^. TEWL was calculated by a Tewameter® (TM300, Courage and Khazaka, Köln, Germany) to determine the water evaporation rate (g/m^2^/h) [16]. A spectrocolorimeter (CD100, Yokogawa, Tokyo, Japan) was used to quantify skin erythema [25, 26].

### 2.8. Histologic Examination of the Skin

The skin specimens were then fixed in buffered formaldehyde solution and sliced into 5 *μ*m sections which were stained with H&E for a histological assessment. Immunohistochemstry staining with 8-hydroxydeoxyguanosine (8-OHdG) and COX-2 (1 : 100 dilution by PBS; Santa Cruz) was treated as described in a previous study [16, 27]. The histological changes were evaluated by using optical microscopy (Olympus BX51, Tokyo, Japan) in nonconsecutive, randomly chosen histological fields. The digital photomicrographs were then processed with DP-72. Image-Pro® plus 4.5 (Media Cybernetics, Bethesda, MD) image analysis software was used to quantify image signals according to a modified version of a protocol described by McGinley and Thompson [28].

### 2.9. Two-Dimensional Electrophoresis (2-DE)

The smashed skin powder was immersed with extraction buffer (7 M urea, 2 M thiourea, 4% CHAPS, 65 mM DTT, and 1 mM PMSF) to homogenize and centrifuge the sample at 10,000 *g* for 20 min at 4°C (KUBOTA 3500, Japan). The concentration of the supernatant was measured by using the Bradford Protein Assay Kit. Protein (200 *μ*g) was solubilized in IPG buffer containing 7 M urea, 2 M thiourea, 4% CHAPS, 65 mM DTT, and 1% IPG buffer to a volume of 350 *μ*L. The samples were then separated by the Immobiline Drystrip (pH 4–7, 18 cm IPG strip, GE Healthcare) on the IPGphor III System for the first dimension. The 2-DE was carried out on 10% acrylamide gels (PROTEAN II XL, Bio-Rad, Hercules, CA, USA) at 30 mA/gel. All gels were visualized by silver staining and then scanned using an Imagescanner (GE Healthcare) [22, 29]. All experiments were repeated three times to confirm the reproducibility.

### 2.10. 2D-Oxyblot

Following IEF, IPG strips were placed in 15 mL test tubes and incubated in 2 N HCl with DNPH (10 mM) at 25°C for 20 min. After the incubation, samples were washed with 2 M Tris-Base/30% glycerol for 15 min. The protein was separated according to molecular weight as described above. 2-DE gels were transferred to a PVDF membrane which was incubated overnight at 4°C with the primary antibody solution consisting of a 1 : 16,000 dilution of the primary antibody (Molecular Probes) in TBST buffer containing 5% milk. The blots were washed and incubated with goat anti-rabbit IgG-conjugated HRP for 2 h. Enhanced chemiluminescence (Immobilon Western Chemiluminescent AP substrate, Millipore) was used for detection [29].

### 2.11. In-Gel Digestion of Proteins and Mass Spectrometric (MS) Analysis

Spots of interest were excised and in-gel digested with trypsin according to previously described procedures [22]. Monoisotopic peptide masses were assigned and used for database searches with the MASCOT search engine (http://www.matrixscience.com) (Matrix Science, London). Search parameters were set as follows: a maximum allowed peptide mass error of 50 ppm and consideration of one incomplete cleavage per peptide.

### 2.12. Statistical Analysis

All values are presented as the mean ± standard deviation (SD). The statistical analysis of the mean values was carried out with the ANOVA using SPSS software [SPSS Inc., Chicago, IL, USA].

## 3. Results

### 3.1. Cell Viability after UVB Exposure under Pretreatment of Different Concentrations of RBE

**Figure 1:**
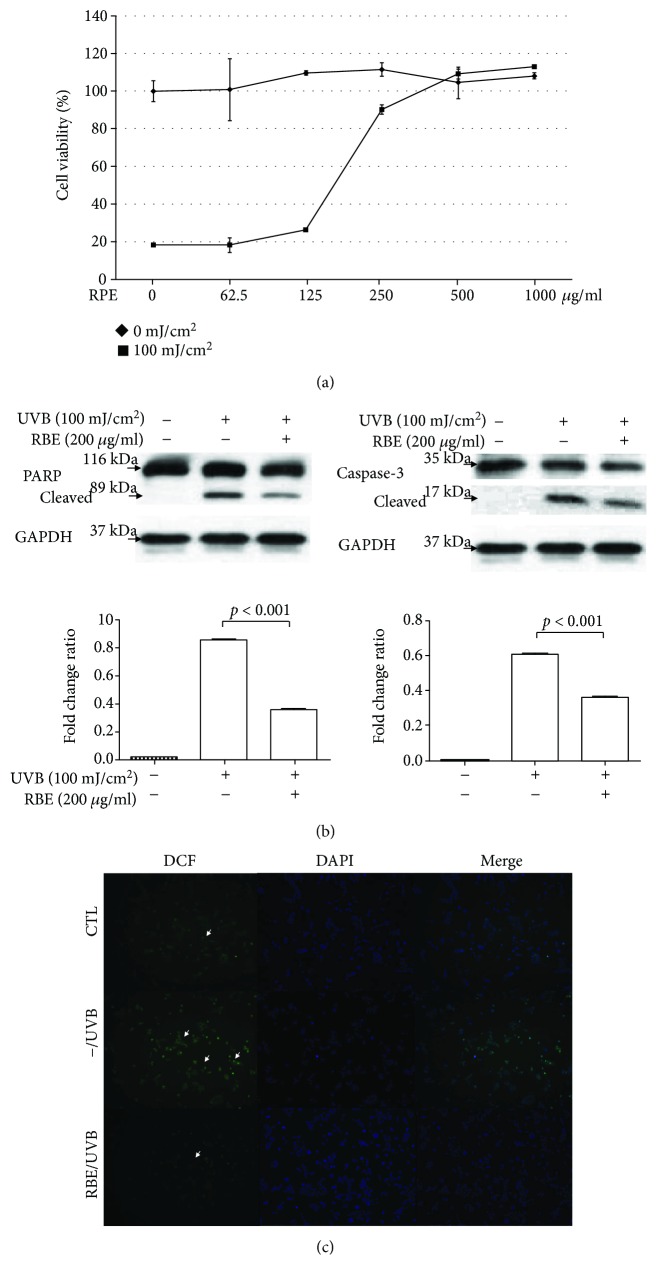
(a) Effects of red raspberry extract on keratinocyte viability with (square) or without (diamond) 100 mJ/cm^2^ UVB exposure as measured by the MTT assays. The cells were applied with different concentrations of red raspberry extract (*x*-axis). Data were the mean ± SD of three independent experiments. (b) PARP and caspase-3 and their cleaved forms were detected by Western blot analysis. GAPDH was used as an internal control. The quantified results were presented by the bar chart. The caspase-3 images were cropped from different parts and exposures of the same gel. (c) Cells were incubated with or without RBE and irradiated by 100 mJ/cm^2^ UVB. The DCF fluorescence was observed under a fluorescence microscope and indicated by arrows.

To evaluate the pharmaceutical effects of red raspberry extract *in vitro*, cell viability was determined by MTT assays. Different concentrations of RBE were applied to the HaCaT cells that were then exposed to 0 or 100 mJ/cm^2^ UVB radiation. As illustrated in Figure 1(a), the results showed that RBE application could effectively attenuate the cell death caused by the UVB exposure in a dose-dependent manner. The EC_50_ of the RBE was 150 *μ*g/mL. Next, we determined the signaling marker proteins, caspase-3, and PARP, with Western blot analysis to further validate the effect of RBE on cell apoptosis. As indicated in Figure 1(b), active forms of caspase-3 (17 kDa) and cleaved PARP (89 kDa) were significantly increased under exposure to 100 mJ/cm^2^ UVB compared to the control whereas administration of RBE could effectively attenuate UVB-induced cell apoptosis, which was accompanied by more moderate cleavage of caspase-3 as well as PARP. Accordingly, UVB irradiation might induce oxidative stress that results in skin cell apoptosis. Dichlorofluorescein (DCF) fluorescent intensity showed that UVB treatment obviously promoted intracellular ROS production compared with the control group within 6 h whereas RBE application alleviated the DCF signal (Figure 1(c)).

### 3.2. Identification of Pure Compounds from RBE

**Figure 2:**
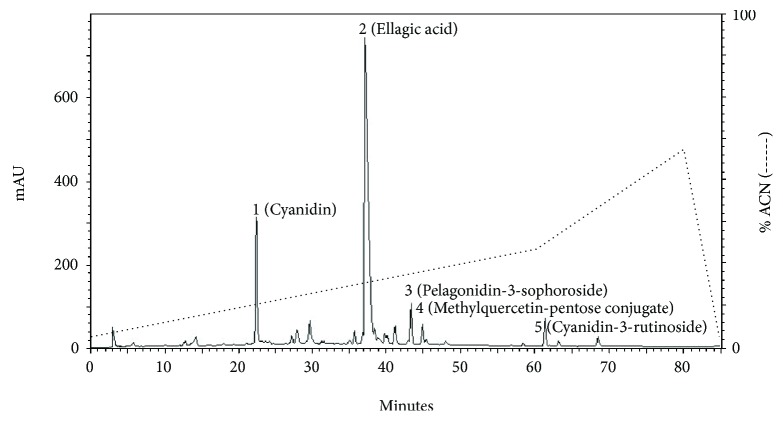
HPLC − UV_*λ*=365 nm_ chromatograms of ethanol extract produced from red raspberry. The quantification of samples was preformed using a HP1100 series HPLC system comprising a gradient pump, and the column used the Agilent Hypersil BDS-C_18_, maintained at ambient room temperatures. 1: cyanidin; 2: ellagic acid; 3: pelagonidin-3-sophoroside; 4: methylquercetin-pentose conjugate; 5: cyanidin-3-rutinoside.

The ethanol-extracted solution was then concentrated to generate brown syrup. The filtered, sterile extract was applied in the subsequent experiments. A high-performance liquid chromatographic method coupled with ultraviolet (UV) was conducted for qualitative determination of the compounds in the RBE. The main compounds contained in RBE were identified by comparing the retention time with the reference standard as follows: cyanidin, ellagic acid, pelagonidin-3-sophoroside, methylquercetin-pentose conjugate, and cyanidin-3-rutinoside (Figure 2).

### 3.3. Effects of RBE upon UVB-Induced Skin Damage

**Figure 3:**
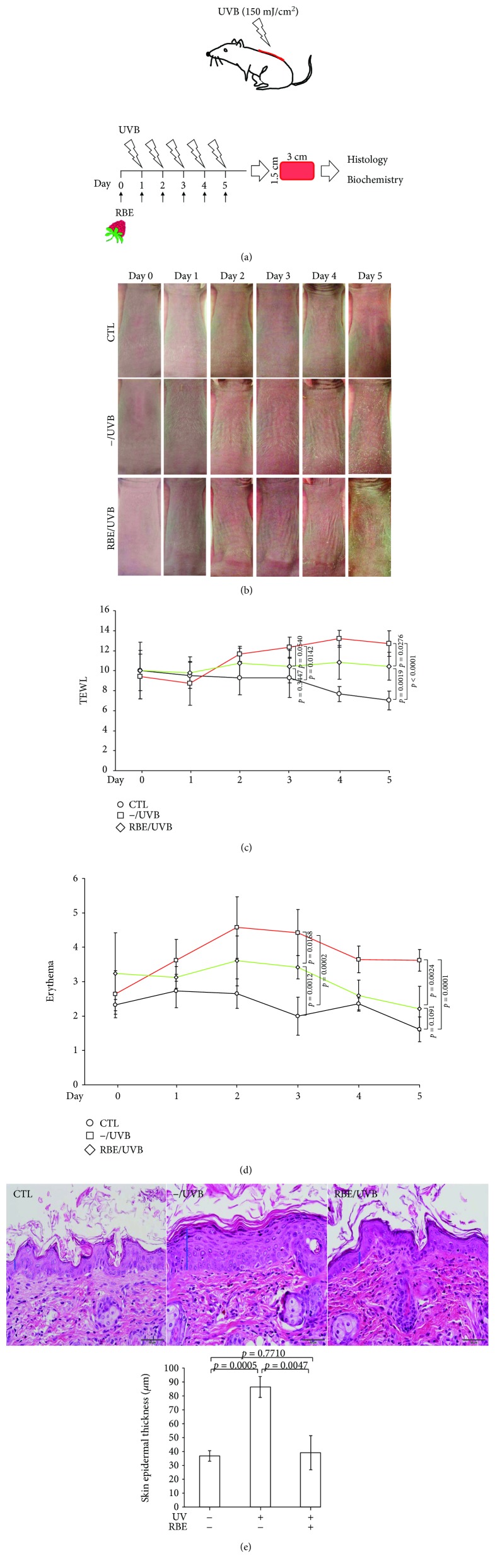
(a) The red raspberry extract was pipetted on a sheet made of nonwoven polyethylene (1.5 × 1.5 cm), and this sheet was applied to the dorsal region of nude mice. Then, the mice were irradiated with UVB (150 mJ/cm^2^) for continuous 5 days and sacrificed. (b) The changes of nude mouse skin quality under different treatments including control (−/−), UVB exposure only (UVB/−), and red raspberry extract application followed by UVB exposure (UVB/R). (c) The effect of red raspberry extract against water loss represented by TEWL. Error bars: mean ± SD. (d) The effect of red raspberry extract against erythema and edema. Error bars: mean ± SD. (e) Histological analysis and assessment of nude mouse skin epidermal thickness from control (CTL), UVB exposure only (−/UVB), and red raspberry extract application followed by UVB exposure (RBE/UVB). Upper panels: H&E staining. Original magnification: 100x. Lower panels: the quantified intensity was indicated by the bar chart. Results represent the mean ± SD of three independent experiments.

UVB radiation (150 mJ/cm^2^) was applied to the back of the mice once a day for 5 days. Prior to UVB exposure, the experimental animals were pretreated with or without 750 *μ*g/mL RBE (Figure 3(a)). After a 5-day irradiation course, the control skin exhibited a flat surface and showed no remarkable wrinkle formation whereas significant wrinkling and scaling were observed in the mouse directly exposed to UVB (Figure 3(b)). As expected, administration of RBE before UVB exposure could effectively prevent the skin injury from the UVB irradiation, which was characterized as mild wrinkling and a low level of scaling as well as dryness on the skin surface (Figure 3(b)). Transepidermal water loss (TEWL) was measured to determine the skin barrier function and the baseline TEWL value of the nude mouse skin, which was about 6–8 g/m^2^/h [30]. As indicated in Figure 3(c), UVB application on day 5 resulted in a 150% enhancement of TEWL compared to the control baseline. Pretreatment of RBE caused a much milder TEWL increment (*p* = 0.0276). UVB irradiation also led to a significant promotion in skin erythema and edema compared to the control. Again, RBE application could significantly alleviate UVB-caused erythema in the skin (*p* = 0.0024; Figure 3(d)). On the other hand, UVB irradiation significantly stimulated cell proliferation of epidermal keratinocytes and the epidermal thickness of the control by 2.25-fold. Utilization of RBE could protect the skin from the abnormal phenomenon (Figure 3(e)). As expected, global protein profile also showed the UVB-induced overexpression of keratins K14 and K17, which may enhance hyperproliferation of keratinocytes (Supplement Figure 1). These findings imply that pretreatment of RBE could minimize various forms of skin damage caused by UVB irradiation.

### 3.4. Antioxidant Ability of RBE in UVB-Irradiated Skin

**Figure 4:**
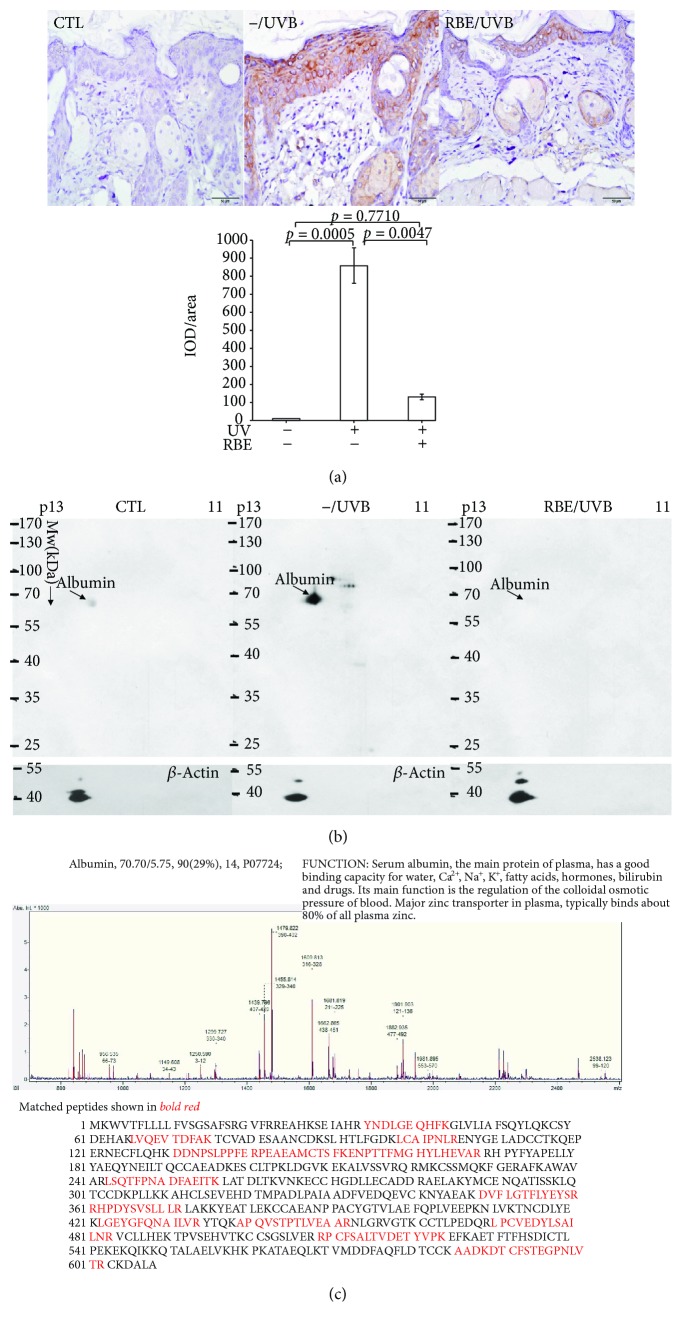
(a) 8-OHdG levels were measured by immunocytochemistry, and the positive cells are demonstrated by brown color staining. The quantified results were indicated by the bar chart. (b) Levels of protein carbonylation. Significantly increased expression of carbonylated proteins were observed in the UVB-exposed group compared to the control, while red raspberry extract could obviously reduce the levels of carbonylated proteins. *β*-Actin was utilized as the loading control. (c) The MALDI-TOF spectrum of trypsinized albumin.

As far as we know, UVB exposure induces the production of oxidative stress, which further attacks DNA and results in 8-OHdG modification. As shown in Figure 4(a), DNA oxidation manifested by the level of 8-OHdG was elicited in the UVB-irradiated skin with respect to that in the control while RBE treatment markedly attenuated the formation of 8-OHdG in the presence of UVB irradiation. Moreover, protein carbonylation is also a critical parameter of oxidative stress.  Figure 4(b) shows that, in the case of the control, protein carbonylation was significantly induced in a UVB-exposed skin. Pretreatment of RBE could effectively prevent the protein oxidation under UVB exposure. The view of oxyblots obtained from the UVB group demonstrates that the extent of oxidation in albumin was dramatically upregulated and there was an obviously decreasing tendency with respect to the protein oxidation in the RBE/UVB group. These results suggest that UVB irradiation directly causes DNA damage and protein oxidation in the skin without the protection of RBE. The equal amount of *β*-actin protein demonstrates that the loading protein volume for all groups is the same. MS analysis was used to unambiguously identify albumin as presented in Figure 4(c).

### 3.5. Signal Transduction Pathways Associated with the Anti-UVB Property of RBE

**Figure 5:**
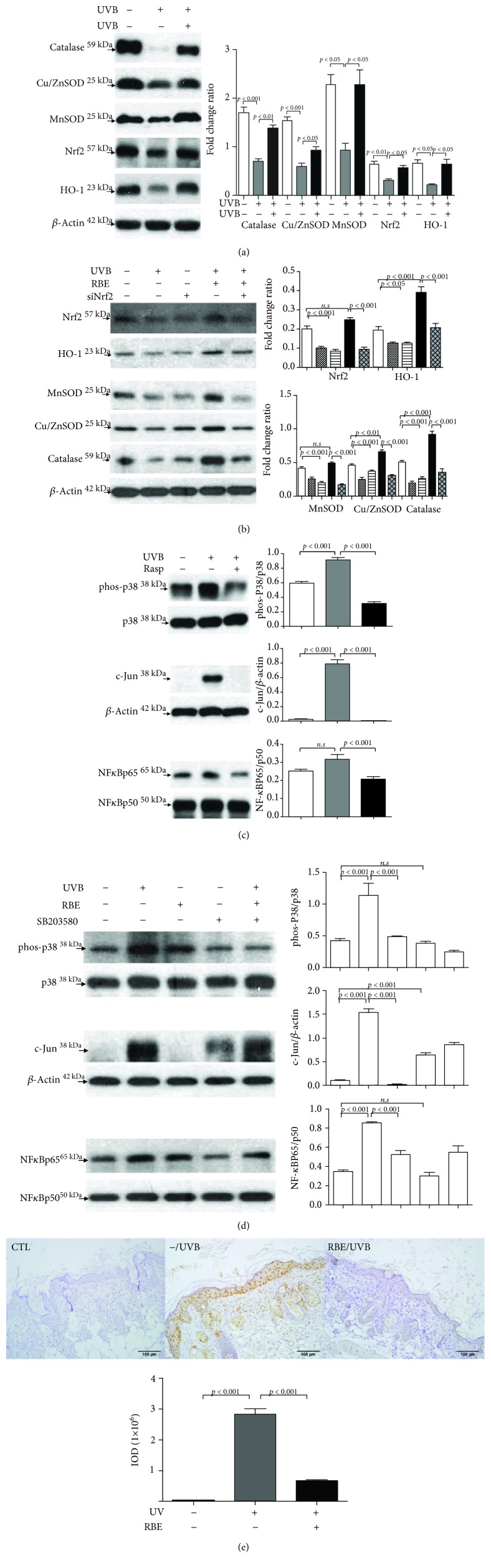
(a) Validation of changes in protein expression after different treatments. Protein levels of catalase, Cu/ZnSOD, MnSOD, Nrf2, and HO-1 were determined by a Western blot analysis. *β*-Actin was used as an internal control. The quantified results were indicated by the bar chart and represent the mean ± SD of three independent experiments. The images were cropped from different gels. (b) HaCaT cells transfected with Nrf2 siRNA were treated with RBE and then exposed to UVB irradiation. Protein levels were measured by Western blot analysis. *β*-Actin was used as an internal control. The quantified results were indicated by the bar chart. (c) Western blot analysis for phosphorylation and total protein levels with different treatments. The phosphorylation levels were normalized by total protein levels. *β*-Actin was applied as the internal control. The images were cropped from different gels. (d) HaCaT cells were preincubated with or without SB203580 for 1 h, irradiated with UVB, and then treated with or without RBE for 6 h. The phosphorylation of p-38 as well as the protein levels of c-Jun and NF-*κ*B subunits (p65 and p50) was determined by specific antibodies. *β*-Actin was applied as the loading control. Quantification of the result was presented as the bar diagram, and the results represent the mean ± SD of three independent experiments. (e) Immunohistochemical staining for the control group (CTL), UVB only group (UVB/−), and RBE/UVB-treated group. The signal with differently expressed cox-2 was shown with brown color. Original magnification: 200x.

Since UVB stimulates the generation of an excessive level of ROS, we then surveyed the content of oxidative stress markers, including catalase and superoxide dismutase (SOD) in different treatments. Decreased catalase and SOD levels were observed in the UVB-applied subjects compared with the control group, while treatments with RBE could enhance the liver catalase and SOD content, protecting the cell against UVB injury. To further reveal the molecular mechanism related to modulation of the antioxidant enzymes, we assessed the critical transcription factor Nrf-2 as a critical contributor to the activation of the antioxidant system. Again, UVB exposure remarkably inhibited Nrf-2 expression with respect to the control, while RBE administration promoted the level of Nrf-2. In line with this finding, a marked decrease in HO-1 expression under UVB exposure was observed. The HO-1 level was significantly restored in response to RBE pretreatment (Figure 5(a)). Moreover, we have utilized Nrf2 RNA interference (siNrf2) to verify RBE-mediated antioxidant ability in a cell model. UVB and siNrf2 significantly reduced the levels of Nrf2, HO-1, and antioxidant enzyme proteins including SOD and catalase while RBE administration could obviously recover the expression of Nrf2, HO-1, and antioxidant systems under UVB exposure (Figure 5(b)). Treatment of the keratinocytes with RBE remarkably attenuated UVB-caused lower expression of Nrf2.

UVB radiation could activate proinflammatory genes that subsequently trigger ROS. In this regard, we next explored the contribution of MAPK family protein p38 and c-Jun signaling to RBE-mediated protection against UVB-induced cell death and inflammatory responses. Our results indicated that the phosphorylation of p38 (p-p38) was significantly upregulated after UVB exposure and pretreatment of RBE obviously suppressed the expression of p-p38 while the total protein level of p38 showed no significant changes under various treatments. Meanwhile, a high level of c-Jun was induced under UVB administration, but exposure to RBE could almost completely inhibit the expression of c-Jun. Moreover, the NF-*κ*B pathway plays a pivotal role in the modulation of gene expression involved in inflammatory responses. The level of NF-*κ*B increased under UVB stimulation while administration with RBE could suppress translocation, activation, and the expression of NF-*κ*B in the mouse skin (Figure 5(c)). Next, we used an upstream signaling p-p38 inhibitor (SB203580) to confirm the molecular mechanism by which RBE inhibits inflammatory cascades induced by UVB. Our results showed that UVB strongly induced the protein expression of p-p38 as well as the inflammatory molecules such as NF-*κ*B and c-Jun. RBE treatment and p-p38 inhibitor could effectively suppress the levels of p-p38, c-Jun, and activated NF-*κ*B in the presence of UVB (Figure 5(d)). These findings implied that RBE could ameliorate p-p38-mediated NF-*κ*B activation and nuclear translocation which will lead to skin inflammatory responses.

Consistently, COX-2 protein expression was evaluated by immunohistochemical analysis to clarify the anti-inflammatory effect of RBE in the skin. As shown in Figure 5(e), COX-2 protein was rarely expressed in the control group. However, the protein expression of COX-2 was markedly augmented upon UVB treatment, and pretreatment of RBE abrogated UVB-induced COX-2 protein expression. These results implied that RBE performs anti-UVB efficacy through modulating the antioxidant and anti-inflammatory signaling pathways.

## 4. Discussion

Exposure to UV radiation, particularly UVB (290–320 nm), elicited harmful biological effects on the skin that could eventually result in histologic and clinical injuries such as skin aging and cancers [31, 32]. Prolonged exposure to UVB radiation would lead to apoptosis of keratinocytes and consequently destroy the skin's natural barrier, thus predisposing the skin to inflammation, infection, and carcinogenesis [33, 34]. Numerous reports have shown that natural product-derived agents would exhibit photoprotective efficacy on a UVB-damaged skin due to their diverse bioactive compounds [35–37]. Moreover, the extract from berries has been added to various skin products such as creams and lotions because the extract is believed to have an efficacy on skin care with a low rate of side effects [38, 39]. In this regard, we investigated the antiphotodamaging activity of RBE both *in vitro* and *in vivo* because the protective potentials of RBE on the skin have remained unresolved.

In our study, the administration of RBE was able to prevent UVB injuries as manifested by the attenuation of cell death of keratinocytes. Meanwhile, animal experiment showed that pretreatment of RBE could attenuate the skin photoaging characterized by skin thickening, erythema, wrinkles, dryness, tanning, and histologic changes, including damage to collagen fibers and abnormal growth of keratinocytes. Particularly, a previous study has indicated that the apoptotic dose of UVB is very similar to the minimal erythema dose (MED) of UVB, implying that UV-induced erythema might be an inflammatory response to the appearance of “sunburn cells” such as apoptotic cells in native human epidermis [40]. It clearly suggests that the epidermis displays abnormal proliferation and differentiation after the UVB-caused sunburn, which appears to be crucially correlated with the impact on skin barrier function leading to photoageing or photocarcinogenesis.

The primary compounds of RBE applied here contain various types of antioxidants including cyanidin, ellagic acid, pelagonidin-3-sophoroside, and their derivatives. These bioactive compounds scavenge free radicals, particularly superoxide anions, and therefore may prevent skin injury since the increased oxygen-derived free radical has been suggested as a pivotal factor in UVB-caused skin problems [41–43]. Our findings showed that UVB exposure induced the ROS production that initiates apoptosis of skin cells and stimulates several genes implicated in the apoptotic process such as the caspase-3 signaling pathway. RBE provided protection against UVB-induced death of skin cells by removal of oxidative stress. Again, ROS production will induce damage to cellular macromolecules such as DNA and protein^1^. In the current study, 8-OHdG and protein carbonylation were surveyed as important hallmarks of oxidative stress. Our results indicated that DNA and protein were highly oxidized in the UVB-administrated subjects compared to the control samples while oxidation of protein and DNA characterized by protein carbonylation and 8-OHdG modification was ameliorated by RBE pretreatment, which attenuated skin injury caused by UVB radiation. Oxidative stress caused by the UVB would result in increased ROS generation and reduced antioxidant capacity, leading to a visible deterioration in skin condition. Albumin, with a good binding capacity for water, was destroyed by carbonylated modification, leading to the decrease of TEWL in the UVB-treated mice [44].

In addition, antioxidant enzymes such as catalase and SOD are consumed during oxidative stress. Therefore, the levels of these enzymes could be used as a hallmark of oxidative stress [45, 46]. Our results also showed that the application of RBE significantly inhibits UVB-induced oxidative stress by upregulating the catalase and SOD levels in the photodamaged skin. The aforementioned findings implied that the protective effects of RBE at least partially contribute to its capability in the ROS-scavenging and antioxidant activity after UVB irradiation.

The transcription factor, Nrf2, may serve as a critical regulator responsible for oxidative stress. It is released and translocated to the nucleus where it stimulates the expression of detoxification enzymes and antioxidant proteins [47, 48]. Of the antioxidant enzymes, HO-1 is considered beneficial for removing ROS in different types of cells. Herein, the levels of Nrf2 and HO-1 were significantly diminished by UVB stimulation accompanied by ROS production whereas RBE remarkably inhibited the Nrf2/HO-1 signaling and inflammatory responses indicated by the suppression of c-Jun as well as NF-*κ*B. UVB-mediated COX-2 expression is associated with erythema, and therefore, COX-2 could be a feasible target for preventing photo-inflammation [18, 49, 50]. RBE remarkably suppressed UVB-promoted protein levels of COX-2 *in vivo.* These results provide evidence that RBE inhibits UVB-induced inflammation and injury in the skin and is mediated by Nrf2/HO-1 activation as well as suppression of the NF-*κ*B pathway, thereby promoting its clinical use in skin therapy. Furthermore, RBE seems to play a functional role against UVB-induced damage via suppressing the activation of p38 MAPK kinases induced by UVB irradiation. Previous research indicated that the blockade of the p38 MAPK pathway inhibited the expression of the proinflammatory cytokines and COX-2, which is in line with our findings [51–53]. In addition, we have measured the absorption spectrum of RBE, which shows that RBE could moderately absorb the UV and the ABS is approximately equal to 0.5 (Supplement Figure 2). In this regard, RBE should partially exhibit the anti-UVB effects via the absorption function, which might explain some of the skin protection effects.

**Figure 6:**
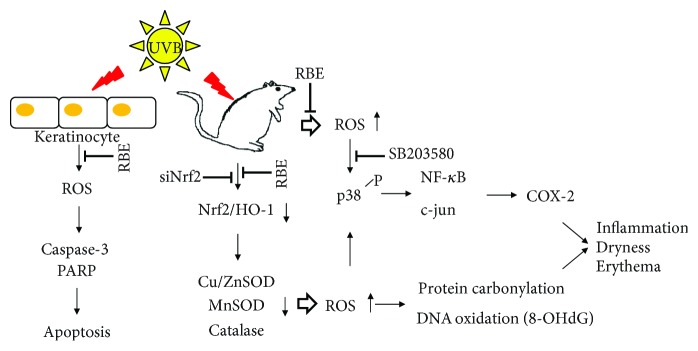
Schematic diagram of UVB-mediated skin injury through suppression of antioxidant enzymes and inducing oxidative modification of biological molecules such as protein and DNA. RBE application could protect the skin against UVB damage via enhancement of antioxidant system as well as inhibition of inflammatory or apoptotic cascades.

In summary, RBE administration protects against UVB-induced photodamage via activating Nrf2 signaling cascade which is referred to as the master regulator of the antioxidant response, modulating various antioxidant enzymes. RBE also inhibits MAPK P38 kinase, c-Jun, and NF-*κ*B to diminish UVB-induced skin inflammation. The RBE may be a promising reagent used in the prevention of photodamage in acute UVB-exposed and chronic inflammatory skin diseases (Figure 6).

## Data Availability

The supplementary data are freely available and can be found in this manuscript.

## Supplementary Materials

Supplementary Materials Supplementary data include typical 2-DE protein profiles of nude mouse skin samples. Close-up pictures indicate the changes in the levels of protein expression among various groups (Supplement Figure 1). Each spot volume was quantified by Prodigy SameSpots software and characterized by MALDI-TOF. Differential expression proteins were listed in the supplement table. Absorption spectrum of RBE in methanol had been shown in Supplement Figure 2.
